# Supplementary material for: Recent wetting trend over Taklamakan and Gobi Desert dominated by internal variability
Source: Nat Commun. 2024 May 23;15:4379. doi: 10.1038/s41467-024-48743-x (PMC11116515; doi:10.1038/s41467-024-48743-x)
Supplement: Supplementary file 1 — Supplementary Information [file 41467_2024_48743_MOESM1_ESM.pdf]

Recent Wetting Trend over Taklamakan and Gobi Desert Dominated by Internal Variability

Wenhao Dong[1,2*], Yi Ming[3], Yi Deng[4], Zhaoyi Shen[5]

[1]Cooperative Programs for the Advancement of Earth System Science, University Corporation for Atmospheric Research, Boulder, CO

[2]NOAA/Geophysical Fluid Dynamics Laboratory, Princeton, NJ

[3]Schiller Institute for Integrated Science and Society and Department of Earth and Environmental Sciences, Boston College, Boston, MA

[4]School of Earth and Atmospheric Sciences, Georgia Institute of Technology, Atlanta, GA

[5]Department of Environmental Science and Engineering, California Institute of Technology, Pasadena, CA

Supplementary Information

This file contains supplementary table and figures: Table S1, Figure S1–Figure S11

Table S1. List of CMIP6 models used in this study along with horizontal resolution (grid numbers) and institution information.

No	Name	Horizontal resolution (lon×lat)	Institution
1	ACCESS-CM2	192×144	Commonwealth Scientific and Industrial Research Organization
2	ACCESS-ESM1-5	192×145	
3	BCC-CSM2-MR	320×160	Beijing Climate Center China Meteorological Administration
4	BCC-ESM1	128×64	
5	CAMS-CSM1-0	320×160	Chinese Academy of Meteorological Science
6	CanESM5	128×64	Canadian Centre for Climate Modeling and Analysis
7	CAS-ESM2-0	256×128	Chinese Academy of Sciences
8	CESM2-FV2	144×96	National Centre for Climate Research
9	CESM2	288×192	
10	CESM2-WACCM-FV2	144×96	
11	CESM2-WACCM	288×192	
12	CIESM	288×192	Tsinghua University
13	CNRM-CM6-1	256×128	Centre National de Recherches Météorologiques
14	CNRM-ESM2-1	256×128	
15	E3SM-1-0	360×180	Lawrence Livermore National Laboratory
16	E3SM-1-1-ECA	360×180	
17	E3SM-1-1	360×180	
18	EC-Earth3	512×256	European EC-EARTH consortium
19	FGOALS-f3-L	288×180	University of Chinese Academy of Sciences
20	FIO-ESM-2-0	288×192	First Institute of Oceanography
21	GFDL-CM4	288×180	NOAA/Geophysical Fluid Dynamics Laboratory
22	GFDL-ESM4	288×180	
23	GISS-E2-1-G	144×90	Goddard Institute for Space Studies
24	GISS-E2-1-H	144×90	
25	INM-CM4-8	180×120	Russian Academy of Science
26	INM-CM5-0	180×120	
27	IPSL-CM6A-LR	144×143	L'Institut Pierre Simon Laplace
28	KACE-1-0-G	192×144	Korea Meteorological Administration
29	MCM-UA-1-0	96×80	University of Arizona
30	MIROC6	256×128	Japan Agency for Marine-Earth Science and Technology
31	MPI-ESM-1-1-HAM	192×96	Max Planck Institute for Meteorology
32	MPI-ESM1-2-HR	384×192	
33	MPI-ESM1-2-LR	192×96	
34	MRI-ESM2-0	320×160	Meteorological Research Institute of the Japan Meteorological Agency
35	NESM3	192×96	Nanjing University of Information Science and Technology
36	NorESM2-LM	144×96	Norwegian Climate Centre
37	NorESM2-MM	288×192	
38	SAM0-UNICON	288×192	Seoul National University
39	TaiESM1	288×192	Research Center for Environmental Changes
40	UKESM1-0-LL	192×144	Met Office Hadley Centre

Supplementary Figures

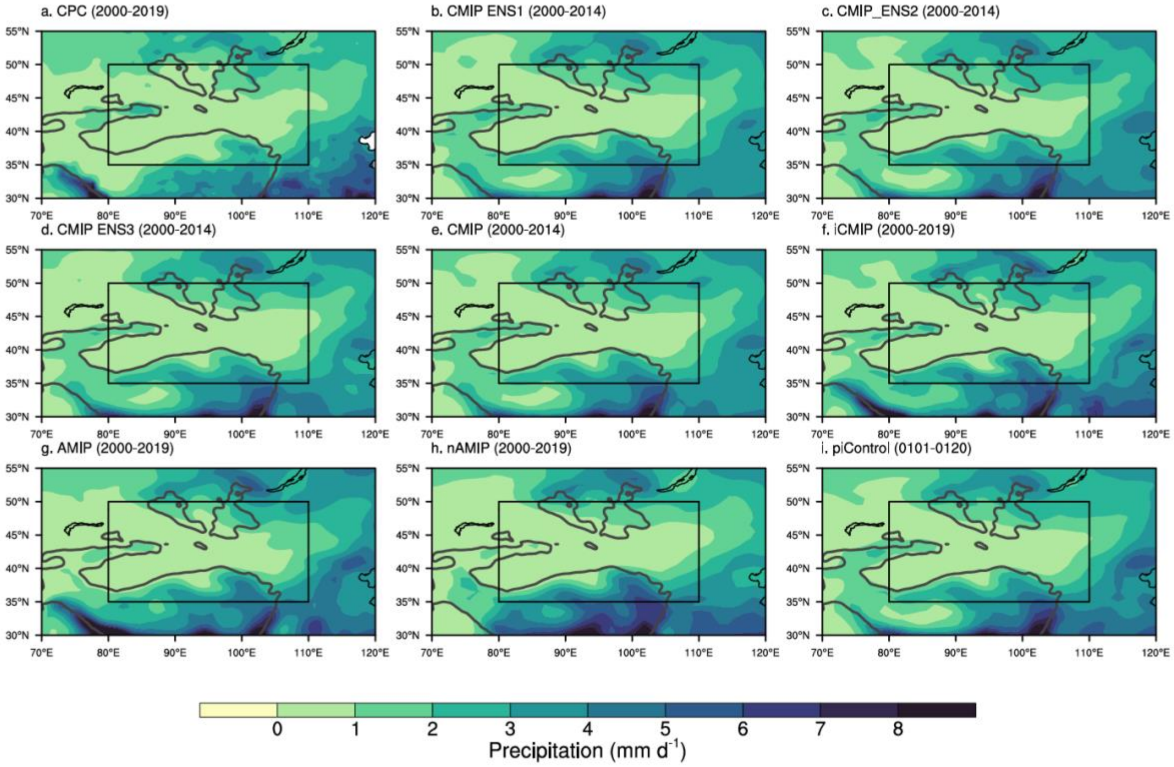

Fig S1. Long-term mean of summer precipitation. Long-term mean of summer precipitation (units: mm d^{-1}) over the Taklamakan and Gobi Desert (TGD) region based on NOAA/Climate Prediction Center (CPC) dataset (a) and different model experiments with their name listed in top-left corner (b-i). The TGD region is delineated by the black rectangle. The gray isoline represents the 2,000-m contour of surface elevation. Source data are provided as a Source Data file.

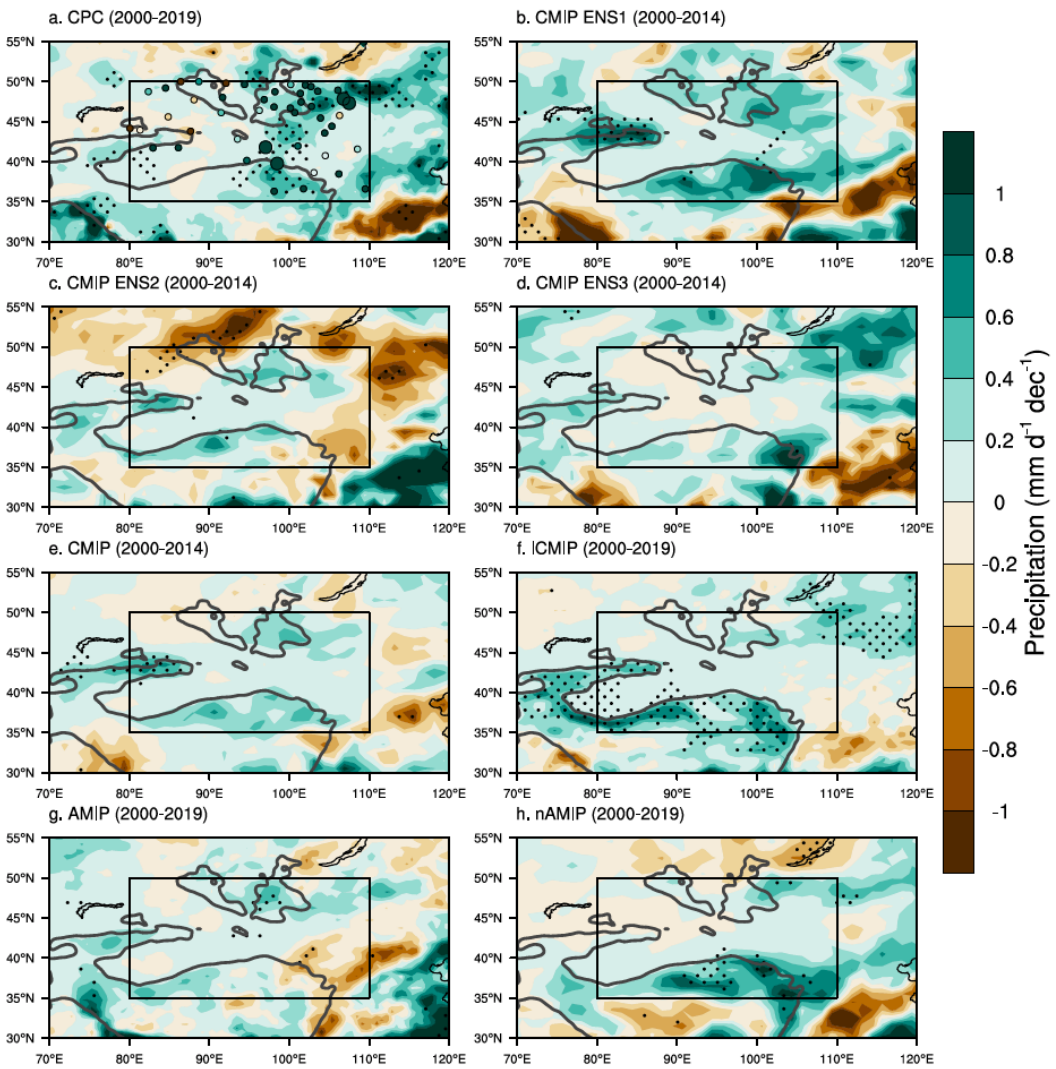

Fig S2. Linear trends of summer precipitation. Linear trends of summer precipitation (units: mm d^{-1} dec^{-1}) based on NOAA/Climate Prediction Center (CPC) dataset (a) and different model experiments with their name listed in top-left corner (b-h). Stippling in (a)-(h) indicates regions where the trends are statistically significant at the 95% confidence level. Colored circles in (a) are results based on the Global Historical Climatology Network (GHCN) station records. The bigger circles indicate the trends are statistically significant at the 95% confidence level. The Taklamakan and Gobi Desert (TGD) region is delineated by the black rectangle. The gray isoline represents the 2,000-m contour of surface elevation. Source data are provided as a Source Data file.

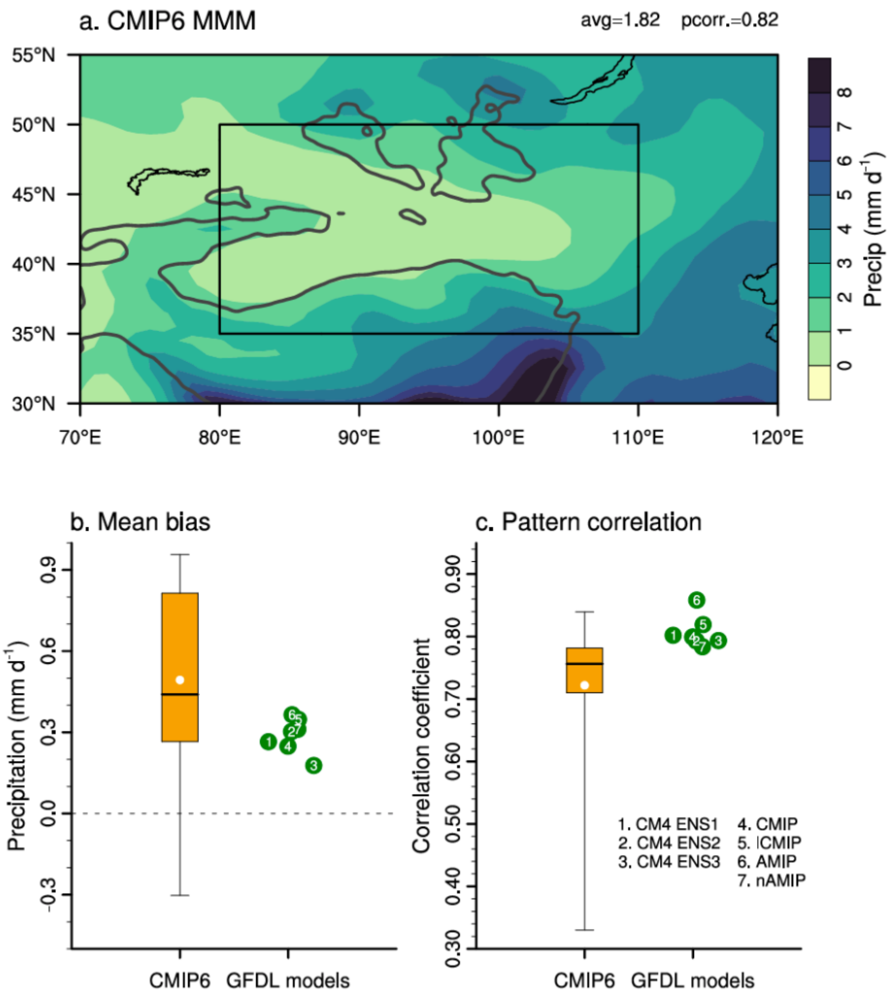

Fig. S3. Simulation of summer precipitation in CMIP6 models. a, Long-term mean of summer precipitation (units: mm d^{-1}) based on CMIP6 multi-model mean. The Taklamakan and Gobi Desert (TGD) region is delineated by the black rectangle. The gray isoline represents the 2,000-m contour of surface elevation. b, Boxplot of mean bias of summer precipitation averaged over the TGD region based on 40 CMIP6 models. Results from GFDL models used in this study are denoted by green circles and are slightly shuffled horizontally for better visualization. c, Similar to (b), but for the precipitation pattern correlation calculated over the TGD region. Mean bias and pattern correlation are calculated relative to the NOAA/Climate Prediction Center (CPC) precipitation dataset. Source data are provided as a Source Data file.

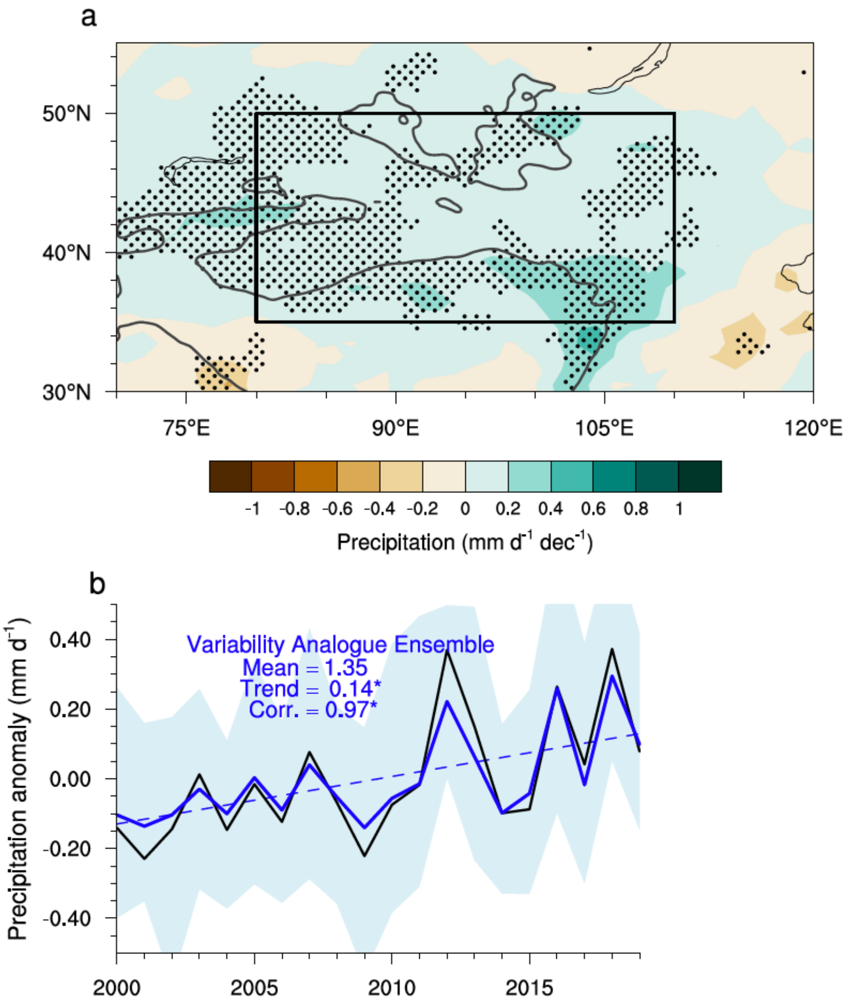

Fig. S4. Composite precipitation trend based on variability analogue ensembles. a, Linear trends of summer precipitation based on the variability analogue ensembles using 5-year segments. Stippling indicates regions where the trends are statistically significant at the 95% confidence level. The Taklamakan and Gobi Desert (TGD) region is delineated by the black rectangle. The gray isoline represents the 2,000-m contour of surface elevation. b, Time series of the summer precipitation averaged over TGD region based on the variability analogue ensembles (blue) and NOAA/Climate Prediction Center (CPC) dataset (black). The light blue shading denotes the spread among the respective ensembles. The mean, linear trend, and the correlation coefficient with the CPC dataset are shown for variability analogue ensembles using 5-year segments. Source data are provided as a Source Data file.

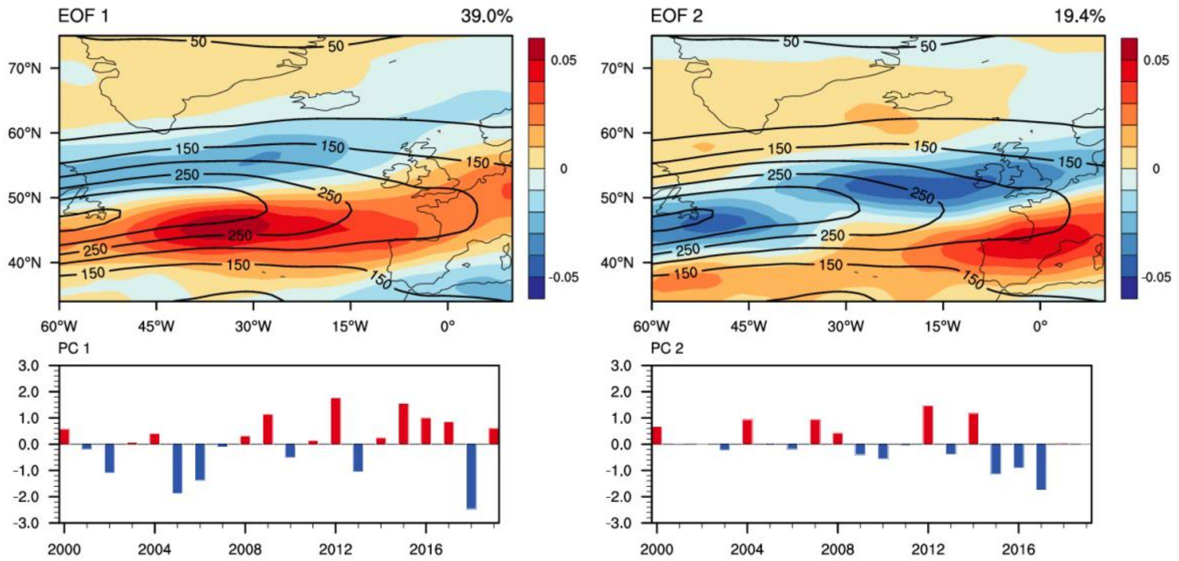

Fig. S5. Empirical Orthogonal Function (EOF) analysis of 200 hPa eddy kinetic energy (EKE) over the North Atlantic. (upper panel), The first and second leading mode pattern of the EOF analysis. Contours denote the 20-year mean EKE (units: $m^2\ s^{-2}$). (bottom panel), The associated principal component (PC1) time series of the first and second leading EOF during 2000–2019. Source data are provided as a Source Data file.

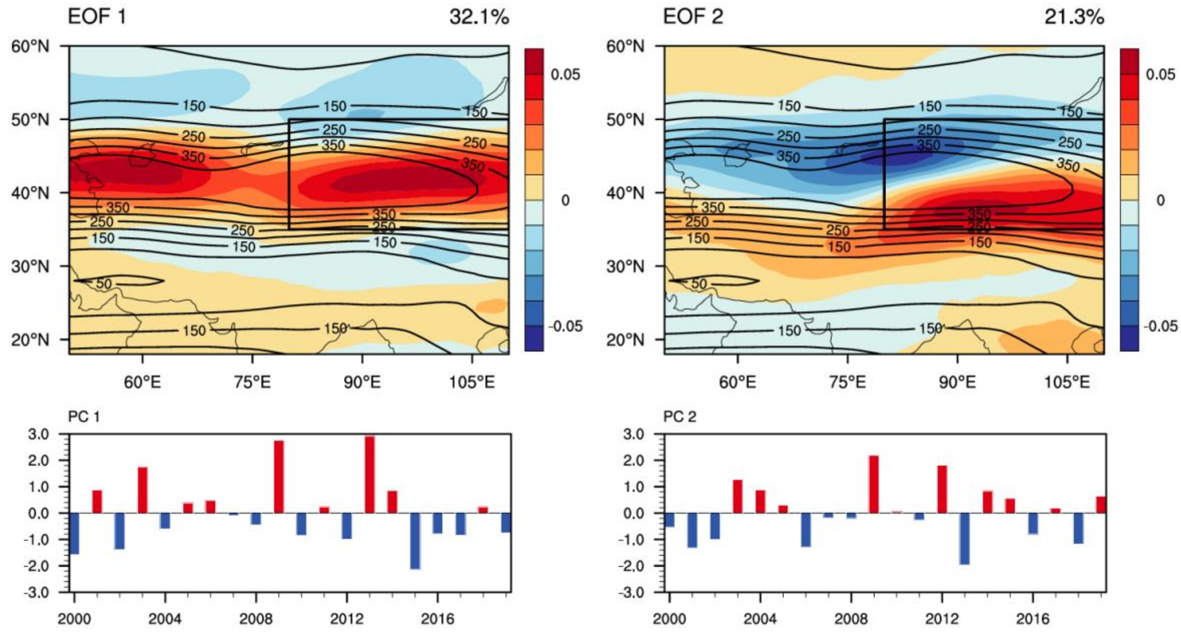

Figure S6. Empirical Orthogonal Function analysis of 200 hPa eddy kinetic energy (EKE) over the Central Asia. (upper panel), The first and second leading mode pattern of the EOF analysis. The Taklamakan and Gobi Desert (TGD) region is delineated by the black rectangle. Contours denote the 20-year mean EKE (units: $m^2\ s^{-2}$). (bottom panel), The associated principal component (PC1) time series of the first and second leading EOF during 2000–2019. Source data are provided as a Source Data file.

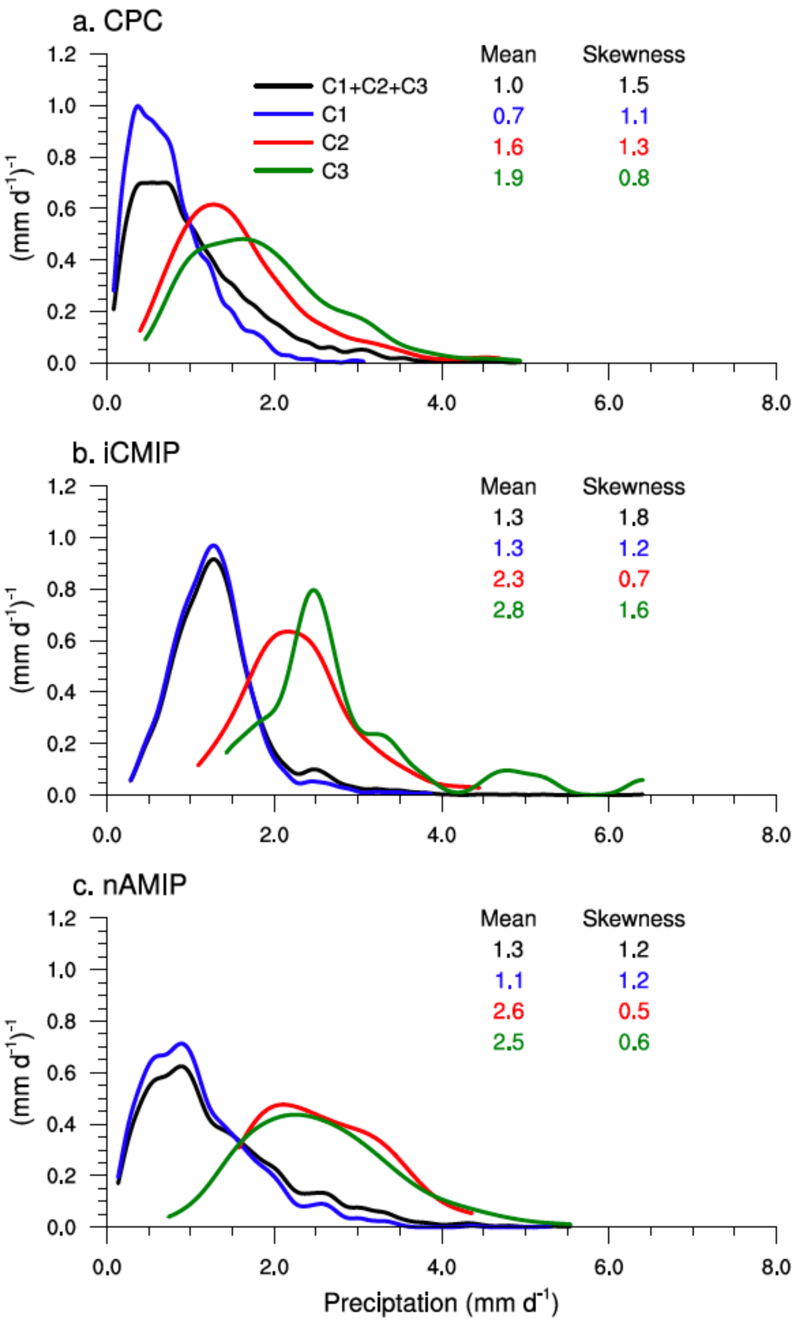

Fig. S7. Precipitation kernel probability density. Kernel probability density estimates of the summer precipitation (units: mm d^{-1}) for each cluster based on (a) the NOAA/Climate Prediction Center (CPC) dataset, (b) iCMIP experiment, and (c) nAMIP experiment. The mean and skewness for each cluster is shown in each panel. Source data are provided as a Source Data file.

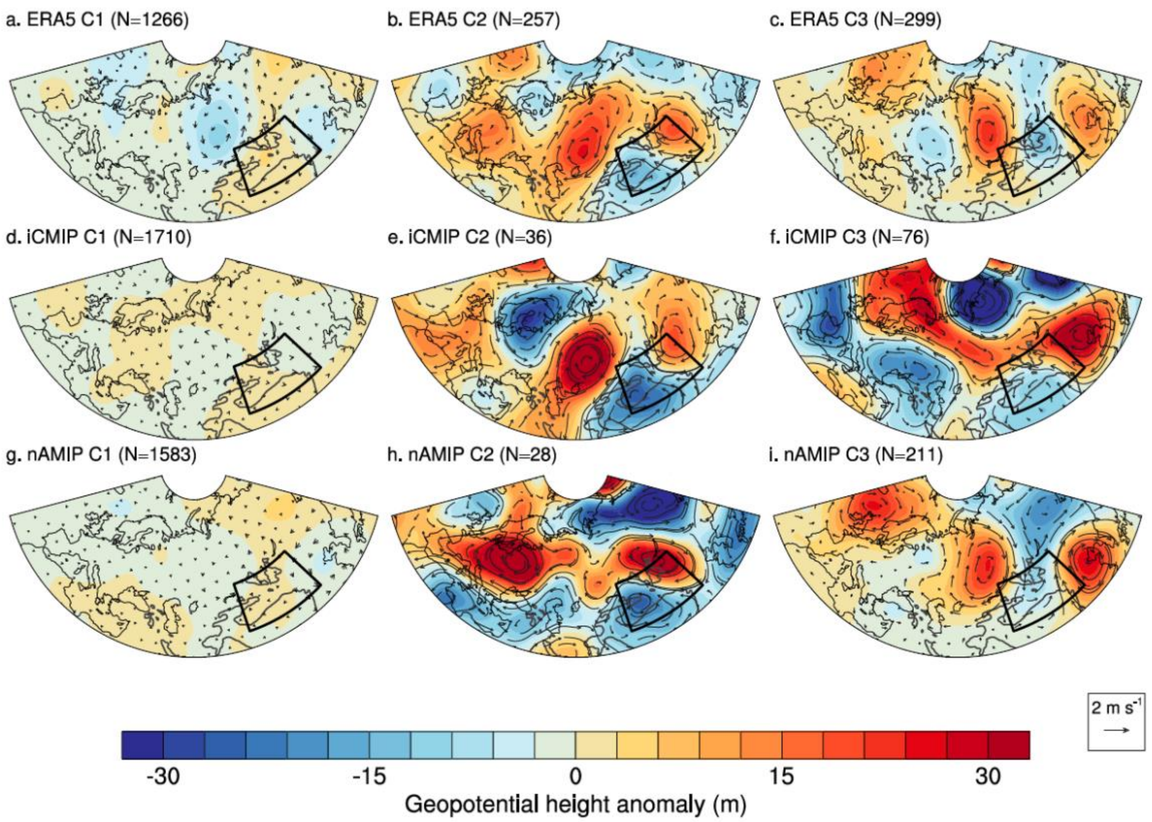

Fig. S8. Mid-level (500 hPa) large-scale circulation patterns. Composite of mid-troposphere (500 hPa) geopotential height anomalies overlaid with horizontal winds for the three clusters based on (a-c) the Fifth Generation of the European Centre for Medium-Range Weather Forecasts (ECMWF) Reanalysis (ERA5), (d-f) iCMIP experiment, and (g-i) nAMIP experiment. The Taklamakan and Gobi Desert (TGD) region is delineated by the black rectangle. The gray isoline represents the 2,000-m contour of surface elevation. Source data are provided as a Source Data file.

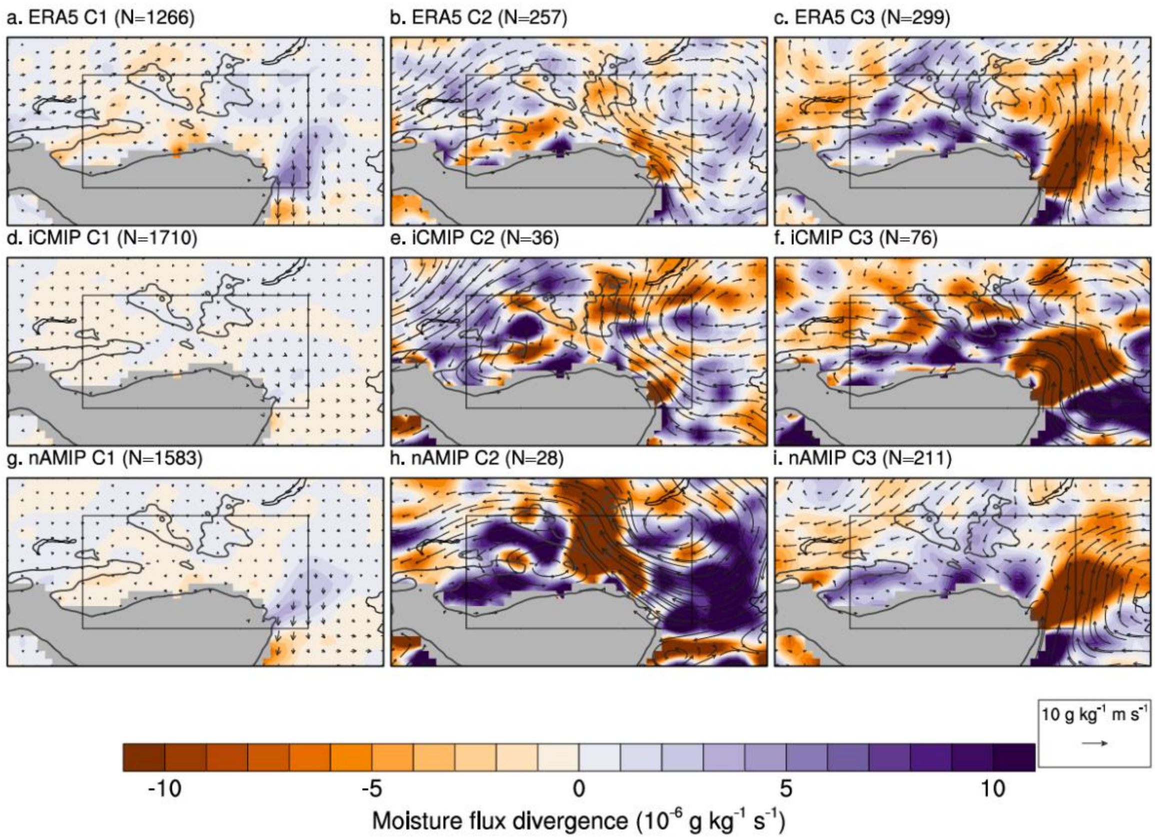

Fig. S9. Low-level (700 hPa) large-scale circulation patterns. Composite of near-surface (700 hPa) moisture flux divergence anomalies overlaid with 700 hPa moisture flux for the three clusters based on (a-c) the Fifth Generation of the European Centre for Medium-Range Weather Forecasts (ECMWF) Reanalysis (ERA5), (d-f) iCMIP experiment, and (g-i) nAMIP experiment. The Taklamakan and Gobi Desert (TGD) region is delineated by the black rectangle. The gray isoline represents the 2,000-m contour of surface elevation. Gray areas indicate region where the 700 hPa surface lies below ground level. Source data are provided as a Source Data file.

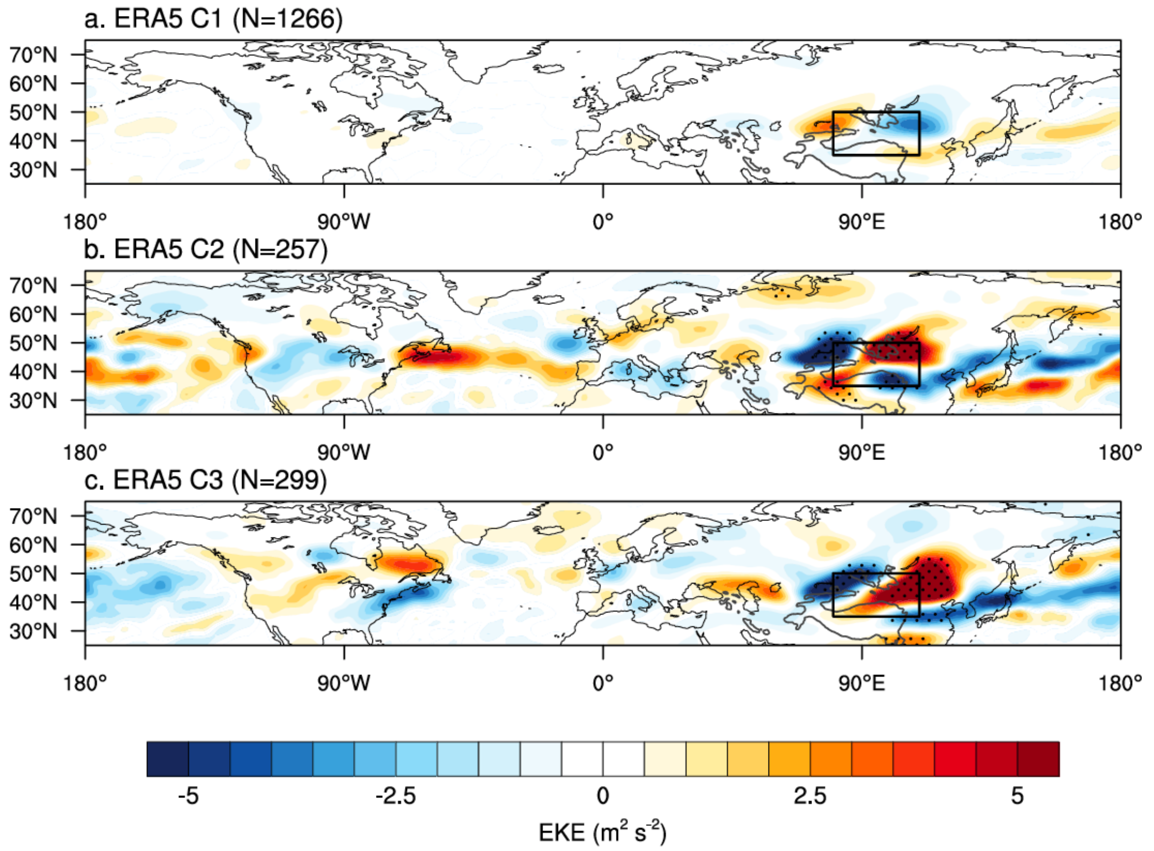

Fig. S10. Eddy kinetic energy (EKE) composite. Composite EKE anomalies for the three clusters based on the Fifth Generation of the European Centre for Medium-Range Weather Forecasts (ECMWF) Reanalysis (ERA5). Dots in (a)-(c) indicate regions where the anomalies are statistically significant at the 95% confidence level. The Taklamakan and Gobi Desert (TGD) region is delineated by the black rectangle. The gray isoline represents the 2,000-m contour of surface elevation. Source data are provided as a Source Data file.

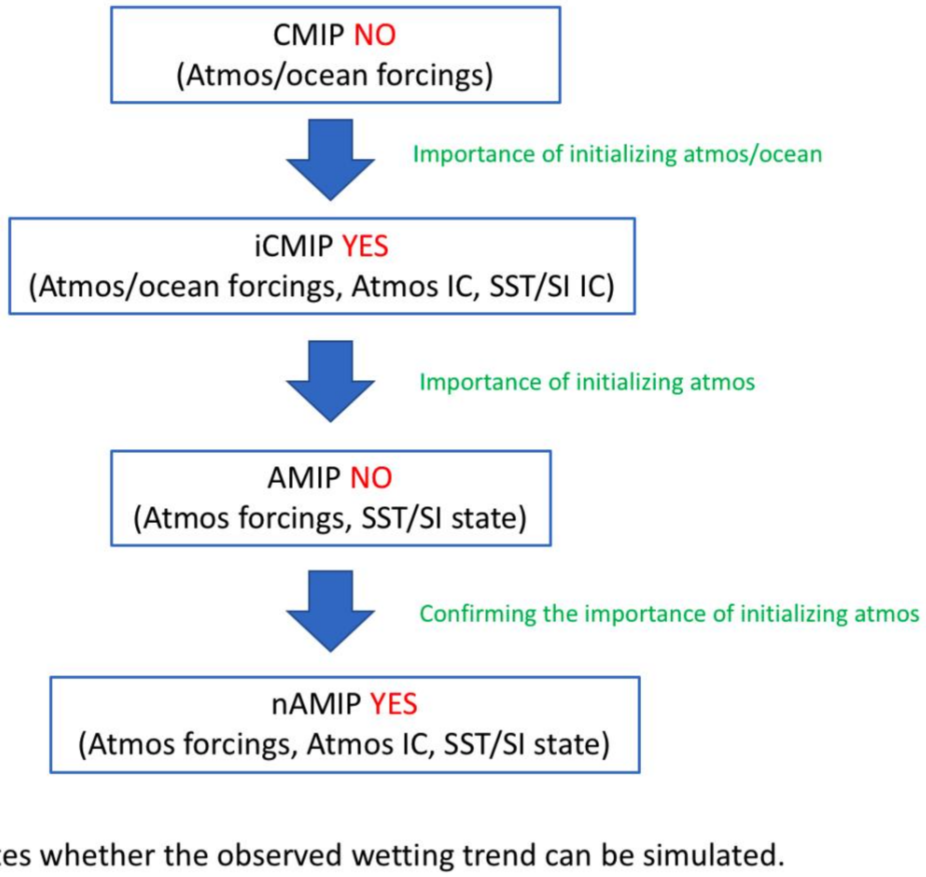

Fig. S11. A schematic of the hierarchical model simulations used in this study. ‘SST/SI state’ indicates that the underlying sea surface temperature (SST) and sea ice (SI) are prescribed, while ‘IC’ denotes the initial conditions for the atmosphere or ocean.
