# Supplementary material for: Recent wetting trend over Taklamakan and Gobi Desert dominated by internal variability
Source: Nat Commun. 2024 May 23;15:4379. doi: 10.1038/s41467-024-48743-x (PMC11116515; doi:10.1038/s41467-024-48743-x)
Supplement: Supplementary file 3 — Reporting Summary [file 41467_2024_48743_MOESM3_ESM.pdf]

nature portfolio

Corresponding author(s): Wenhao Dong

Last updated by author(s): May 5, 2024

Reporting Summary

Nature Portfolio wishes to improve the reproducibility of the work that we publish. This form provides structure for consistency and transparency in reporting. For further information on Nature Portfolio policies, see our Editorial Policies and the Editorial Policy Checklist.

Statistics

For all statistical analyses, confirm that the following items are present in the figure legend, table legend, main text, or Methods section.

n/a	Confirmed	
[ ]	[x]	The exact sample size (*n*) for each experimental group/condition, given as a discrete number and unit of measurement
[ ]	[x]	A statement on whether measurements were taken from distinct samples or whether the same sample was measured repeatedly
[ ]	[x]	The statistical test(s) used AND whether they are one- or two-sided<br>*Only common tests should be described solely by name; describe more complex techniques in the Methods section.*
[x]	[ ]	A description of all covariates tested
[x]	[ ]	A description of any assumptions or corrections, such as tests of normality and adjustment for multiple comparisons
[ ]	[x]	A full description of the statistical parameters including central tendency (e.g. means) or other basic estimates (e.g. regression coefficient) AND variation (e.g. standard deviation) or associated estimates of uncertainty (e.g. confidence intervals)
[ ]	[x]	For null hypothesis testing, the test statistic (e.g. *F*, *t*, *r*) with confidence intervals, effect sizes, degrees of freedom and *P* value noted<br>*Give P values as exact values whenever suitable.*
[x]	[ ]	For Bayesian analysis, information on the choice of priors and Markov chain Monte Carlo settings
[x]	[ ]	For hierarchical and complex designs, identification of the appropriate level for tests and full reporting of outcomes
[ ]	[x]	Estimates of effect sizes (e.g. Cohen's *d*, Pearson's *r*), indicating how they were calculated

Our web collection on statistics for biologists contains articles on many of the points above.

Software and code

Policy information about availability of computer code

Data collection	The datasets used in this study were obtained directly from public access sources or generated by climate models, with specific details provided in the Data Availability statement.
Data analysis	The NCAR Command Language (NCL v6.6.2) is used for plotting. All custom codes are direct implementations of standard methods and techniques, described in detail in Methods.

For manuscripts utilizing custom algorithms or software that are central to the research but not yet described in published literature, software must be made available to editors and reviewers. We strongly encourage code deposition in a community repository (e.g. GitHub). See the Nature Portfolio guidelines for submitting code & software for further information.

Data

Policy information about availability of data

All manuscripts must include a data availability statement. This statement should provide the following information, where applicable:

- Accession codes, unique identifiers, or web links for publicly available datasets
- A description of any restrictions on data availability
- For clinical datasets or third party data, please ensure that the statement adheres to our policy

The monthly Tropical Rainfall Measuring Mission (TRMM) precipitation data is accessible at https://disc.gsfc.nasa.gov/datasets/TRMM_3B43_7/summary. The station records of the monthly precipitation dataset are available for acquisition at https://www.ncei.noaa.gov/data/ghcnm/v4beta/. The other precipitation datasets utilized in this study, including the CPC Global Unified Gauge-Based Analysis of Daily Precipitation dataset, the monthly GPCP precipitation dataset, the

monthly UDel precipitation dataset, and the monthly CPC Merged Analysis of Precipitation (CMAP) precipitation dataset, can be found and downloaded at https://psl.noaa.gov/data/gridded/index.html. The PC-based summer NAO index is obtained from https://climatedataguide.ucar.edu/sites/default/files/2023-04/nao_pc_monthly.txt. For the ERA5 reanalysis datasets, they can be accessed from https://apps.ecmwf.int/data-catalogues/era5/?class=ea. The GFDL AM4 model source code can be obtained from https://data1.gfdl.noaa.gov/nomads/forms/am4.0/. Model outputs from AMIP, CMIP, and piControl experiments can be downloaded from the CMIP6 data portal (https://aims2.llnl.gov/search/cmip6/). Model outputs from iCMIP experiment can be accessed publicly from the GFDL SPEAR Large Ensembles website (https://noaa-gfdl-spear-large-ensembles-pds.s3.amazonaws.com/index.html#SPEAR/GFDL-LARGE-ENSEMBLES/CMIP/NOAA-GFDL/GFDL-SPEAR-MED/historical/). Model outputs from the nAMIP experiment have been deposited in the Zenodo database under accession code (https://doi.org/10.5281/zenodo.11110869). Source data are provided with this paper.

Research involving human participants, their data, or biological material

Policy information about studies with human participants or human data. See also policy information about sex, gender (identity/presentation), and sexual orientation and race, ethnicity and racism.

Reporting on sex and gender	NA
Reporting on race, ethnicity, or other socially relevant groupings	NA
Population characteristics	NA
Recruitment	NA
Ethics oversight	NA

Note that full information on the approval of the study protocol must also be provided in the manuscript.

Field-specific reporting

Please select the one below that is the best fit for your research. If you are not sure, read the appropriate sections before making your selection.

☐ Life sciences ☐ Behavioural & social sciences ☒ Ecological, evolutionary & environmental sciences

For a reference copy of the document with all sections, see nature.com/documents/nr-reporting-summary-flat.pdf

Ecological, evolutionary & environmental sciences study design

All studies must disclose on these points even when the disclosure is negative.

Study description	NA
Research sample	NA
Sampling strategy	NA
Data collection	NA
Timing and spatial scale	NA
Data exclusions	NA
Reproducibility	NA
Randomization	NA
Blinding	NA

Did the study involve field work? ☐ Yes ☒ No

Reporting for specific materials, systems and methods

We require information from authors about some types of materials, experimental systems and methods used in many studies. Here, indicate whether each material, system or method listed is relevant to your study. If you are not sure if a list item applies to your research, read the appropriate section before selecting a response.

Materials & experimental systems

n/a	Involved in the study
☒	☐ Antibodies
☒	☐ Eukaryotic cell lines
☒	☐ Palaeontology and archaeology
☒	☐ Animals and other organisms
☒	☐ Clinical data
☒	☐ Dual use research of concern
☒	☐ Plants

Methods

n/a	Involved in the study
☒	☐ ChIP-seq
☒	☐ Flow cytometry
☒	☐ MRI-based neuroimaging

Plants

Seed stocks	NA
Novel plant genotypes	NA
Authentication	NA
